# Molecular Analysis of HLA-G in Women with High-Risk Pregnancy and Their Partners with Regard to Possible Complications

**DOI:** 10.3390/ijerph16060982

**Published:** 2019-03-19

**Authors:** Olimpia Sipak, Aleksandra Rył, Anna Grzywacz, Maria Laszczyńska, Sławomir Szymański, Beata Karakiewicz, Iwona Rotter, Cezary Cybulski

**Affiliations:** 1 Department of Obstetrics and Pathology of Pregnancy, Pomeranian Medical University in Szczecin, 71-210 Szczecin, ul. Żołnierska 48, Poland; slawomir.szymanski@pum.edu.pl; 2 Department of Medical Rehabilitation and Clinical Physiotherapy, Pomeranian Medical University in Szczecin, 71-210 Szczecin, ul. Żołnierska 48, Poland; aleksandra.ryl@pum.edu.pl (A.R.); iwrot@wp.pl (I.R.); 3 Independent Laboratory of Health Promotion, Pomeranian Medical University in Szczecin, 70-103 Szczecin, ul. Gen. Dezyderego Chłapowskiego 1, Poland; anna.grzywacz@pum.edu.pl; 4 Department of Histology and Developmental Biology, Pomeranian Medical University in Szczecin, 71-210 Szczecin, ul. Żołnierska 48, Poland; laszcz@pum.edu.pl; 5 Department of Public Health, Pomeranian Medical University in Szczecin; 71-210 Szczecin, ul. Żołnierska 48, Poland; beata.karakiewicz@pum.edu.pl; 6 Department of Genetics and Pathology, Pomeranian Medical University in Szczecin, 71-252 Szczecin, ul. Unii Lubelskiej 1, Poland; cezarycy@pum.edu.pl

**Keywords:** *HLA-G*, allele, haplotype, pregnancy

## Abstract

The understanding of the molecular and biochemical characteristics of the human leukocyte antigen-G (*HLA-G*) is important because of the diverse influence of this antigen’s polymorphisms on the course of a pregnancy. The aim of our study was to assess how the variation of the *HLA-G* allele and the *HLA-G* 14-bp ins/del polymorphism influence predisposition to a complicated pregnancy. The clinical material consisted of parental pairs with complicated pregnancies (210 women; 190 men). The control group included parental pairs without complications during pregnancy (89 women; 86 men). The study involved isolation of genome DNA from peripheral blood leukocytes, sequencing, and analysis of the 14-bp ins/del polymorphism in the 3′-untranslated region (3′-UTR) of the *HLA-G* gene based on polymerase chain reaction (PCR). The most common *HLA-G* allele in the group of women with complicated pregnancies was the *HLA-G 10101* allele. There were no statistically significant differences in the frequencies of the 14-bp ins/del polymorphism in the 3′UTR of the *HLA-G* gene between the groups. Our results suggest that the risk of complications in pregnancy is influenced by the *HLA-G 10101*, *HLA-G 10108*, and *HLA-G 10106* alleles and is not influenced by the 14-bp ins/del polymorphism in the 3′UTR of the *HLA-G* gene.

## 1. Introduction

During embryo implantation and pregnancy, the maternal immune system comes into close contact with fetal trophoblast cells. To avoid rejection of a semiallogeneic fetus, the mechanisms modulating the maternal immune system must be initiated [1].

The understanding of the molecular and biochemical characteristics of the human leukocyte antigen-G (*HLA-G*) and its products is important due to the diverse influence of this antigen’s polymorphisms on the course of the pregnancy. *HLA-G* was the first identified trophoblast HLA, located on the short arm of the 6p21.1–6p.21.3 chromosome. It is a highly polymorphic group, including HLA class Ia genes (*HLA-A*, *-B*, and *-C*) and HLA class Ib genes (*HLA-E*, *-F*, and *-G*) [2]. The roles of the majority of the HLA molecules have already been well documented. HLA class I molecules are located on the surface of all nucleated cells of the body, erythrocytes, and thrombocytes. The expression of MHC class II molecules—responsible for presenting antigens to CD4+ T-helper lymphocytes—is mainly observed on the surface of antigen-presenting cells (APC). This process belongs to the key mechanisms of immune response regulation that enable recognition and effective reaction to foreign antigens, but may also provoke pathological recognition of the body’s own antigens [2,3].

The *HLA-G* polymorphism is the one that seems to be the most essential from the point of view of the course of a pregnancy. Belonging to the nonclassical HLA class I molecules, *HLA-G* has been, for the first time, detected on the surface of trophoblast cells in the early stage of pregnancy. It is believed to be a factor that influences the process of implantation of the fertilized ovum in the mucous membrane of the uterus [4]. *HLA-G* expression is observed in ova immediately after fertilization at the oocyte stage [5]. Under physiological conditions, the *HLA-G* antigen protects fetal tissues against natural killers (NKs) and cytotoxic lymphocytes through inhibiting their activity. Decreased expression of *HLA-G* has been observed under conditions posing a threat to pregnancy, such as preeclampsia and recurrent spontaneous abortion, which confirms its protective effect on pregnancy [6].

It is worth emphasizing that the molecular structure of *HLA-G* has many polymorphic variants, but most of them have no impact on the sequences of emerging amino acids. Nevertheless, there are also mutations contributing to the protein structure; a single base pair (bp) deletion at nucleotide 1597 causes frame-shift to the 130th amino acid. The cytosine deletion on codon 130 results in the creation of the null allele (called G * 0105N) that cannot encode the functional *HLA-G* isoform. It has been demonstrated that the null allele may be related to a higher risk of recurrent spontaneous abortion [7]. The mutation that is essential for the course of pregnancy is the *HLA-G* gene 14-bp insertion (ins)/deletion (del) (rs66554220) polymorphism in exon 8, which has an impact on the *HLA-G* mRNA stability and isoform splicing patterns [8]. *HLA-G* expression is regulated by some polymorphisms in the 5′-URR (upstream regulatory region) and also a 14-base pair (bp) ins/del in the 3′-untranslated region (3′-UTR). Researchers have reported a significant association between the single nucleotide polymorphism (SNP) at position 1754 in exon 8 in the 3′-UTR (untranslational region) of the *HLA-G* gene and the risk of preeclampsia [9].

The aim of this study was to assess how the variation of the *HLA-G* allele and the *HLA-G* 14-bp ins/del polymorphism in parental pairs influence their predisposition to pregnancy complicated by antiphospholipid syndrome (APS), preeclampsia (PE), intrauterine growth restriction (IUGR), and recurrent spontaneous abortion (RSA).

## 2. Materials and Methods

### 2.1. Experimental Groups and a Control Group

The clinical material consisted of retrospectively assessed parental pairs (211 women aged 20–35 years and their partners—189 men aged 21–42 years) in which women had experienced an unsuccessful or complicated pregnancy. The control group included parental pairs—89 women and their 86 partners—having offspring without complications during pregnancy. All studied pregnant women were Caucasian Poles. Blood samples from the cubital vein were taken from all participants. Blood for laboratory analysis was collected from the women in the experimental and the control groups after the end of pregnancy.

All women involved in the study were patients of the Clinic of Maternal and Fetal Medicine, the Outpatient Clinic at the Clinic of Maternal and Fetal Medicine, and the Rheumatology Outpatient Clinic, Pomeranian Medical University in Szczecin (Poland). The protocol was approved by the Bioethical Commission of the [covered for blind review] (approval number BN-001/94/07). All participants gave voluntary and informed written consent to take part in the study.

### 2.2. Division into Groups

The women involved in the study and their partners were divided into a control group (C) and four experimental groups depending on the course of pregnancy, namely a group with antiphospholipid syndrome, a group with preeclampsia, a group with intrauterine growth restriction, and a group with recurrent spontaneous abortion. The patients were assigned to the groups according to the criteria shown in Table 1.

### 2.3. Isolation of Genome DNA from Peripheral Blood Leukocytes

DNA was isolated using the detergent method. 10-mL peripheral blood samples were collected from the patients, each to be used with 1 mL of 10% edetate disodium. 20 mL of TKM buffer (10 mM Tris–HC1, 10 mM KCI, 2 mM EDTA, 4 mM Mgc1 2) and the detergent IGEPAL (Sigma) were added to degrade cell membranes. Next, the samples were spun for 10 min (3400 rotations per minute, 12 °C). 30 mL and 20 mL of TKM were added one by one to the obtained leukocyte sediment, and spun again. To break the sediment and obtain homogeneous suspension, 2 mL of TKM was added. 0.5 mL of 10% SDS (sodium dodecyl sulphate) was added to the obtained pure leukocyte sediment. Then, it was incubated in a water bath at 60 °C for 7 min until the cell membranes and protein junctions were broken. The protein was salted out with 1 mL of 5 M NaCl solution. The mixture was shaken until homogeneous emulsion was achieved and then spun (9500 rotations per minute, 12 °C). 5 mL of 96% ethanol was added to the obtained supernatant to precipitate the DNA. As the final step, the DNA was rinsed out with 1 mL of ethanol, and dried in a vacuum centrifuge for 10 min. The last stage involved adding 3–4 drops of the TE buffer (10 mM Tris–HCl, 1 mM EDTA). The isolated DNA was diluted to the concentration of 50 ng/µL.

### 2.4. Sequencing

Sequencing of exons 2, 3, and 4 of the *HLA-G* gene was performed (Table 2). Preparative PCR was performed in 25 µL of solution containing 50 ng of the genomic DNA, 2.5 µL 10× reaction buffer (Polgen), 200 µM of each deoxynucleotide, 6 pM of each primer, and 1 U Taq DNA polymerase. Preparative PCR was performed in 35 cycles under the following conditions: Initial denaturation 95 °C, 5 min; denaturation 95 °C, 30 s; primer annealing 55–60 °C, 30–40 s; elongation 72 °C, 30 s; final elongation 72 °C, 5 min.

The amplicons were put on a 100× Microcon column (Amicon), which was placed in an Eppendorf test tube. 400 µL of distilled water was added and the whole thing was spun (1850 G, 15 min). The filtrate was poured out, and the reaction products left on the filter were poured over with 400 µL of water and spun again. This cycle was repeated thrice. To retrieve purified PCR products, the column was put into a new test tube and spun (9000 G, 10 min). The product was diluted with 20 µL of water. Asymmetric PCR was performed in an automatic thermocycler Gene Amp PCR System 9600 (Perkin Elmer) in a reaction mixture containing 4 µL of a purified PCR product, 30 pM primer, 8 µL of Prism Ready Reaction Mix (DyeDeoxy Terminator Cycle Sequencing Kit from Applied Biosystems, Foster City, CA, USA). The parameters of sequential PCR include: Initial denaturation 96 °C, 15 s; 25 cycles: Denaturation 94 °C, 20 s; primer annealing 55 °C, 30 s; DNA elongation 60 °C, 4 min.

The amplicons were precipitated with 96% ethanol, rinsed with 70% ethanol, and dried in a vacuum apparatus Eppendorf Concentrator 5301. Next, they were dissolved in 4 µL of loading buffer (150 µL of deionized formamide, 50 µL of 50 mM EDTA, 0.05% Dextran Blue). The samples were denatured for 4 min at 94 °C, put into an ice water bath, and placed on a denaturing polyacrylamide sequence gel (6% acrylamide—19:1, 1 × TBE, 8 M urea). Electrophoretic separation was performed using a 373A DNA Sequencer (Applied Biosystems). Electrophoresis data collection and analysis were performed using the 373 Data Collection and the 373 Analysis software (Applied Biosystems).

The *HLA-G* alleles observed in our study were determined by comparison of the base pair sequences in exons 2, 3, and 4 and the base pair sequences in the *HLA-G* alleles published on the website of Nolan Institute [14].

### 2.5. Analysis of the 14-bp ins/del Polymorphism in the 3′-Untranslated Region of the HLA-G Gene

The polymorphic region was amplified by polymerase chain reaction (PCR). The PCR amplicons were resolved on 4% agarose gel. The insertion allele was 14 nucleotides longer than the deletion allele. The PCR reaction was performed in a thermocycler (GeneAmp PCR System 9700, Applied Biosystems). The following primers were used in the PCR reaction: F 5′GTGATGGGGCTGT1T MAGTGTCACC 3′/R 5′GGMGGAATGCAGJTCAGCATGA 3′. The reaction was performed under the following conditions: Preliminary denaturation at 94 °C, 5 min; 33 cycles: Denaturation at 94 °C, 25 s; primer annealing 68 °C, 35 s; elongation 72 °C, 35 s.

### 2.6. Statistical Analysis

Statistical analysis was performed using Statistica v. 17.0. The chi-square test was employed to verify whether the genotype frequencies fit the Hardy–Weinberg equilibrium (H-W). The discrete variables were assessed by Pearson’s chi-square test. The logistic regression model was used to estimate the risk of antiphospholipid syndrome, preeclampsia, intrauterine growth restriction, and recurrent spontaneous abortion for particular alleles of the 14-bp ins/del polymorphism in the 3′-UTR of the *HLA-G* gene. The results were described in terms of a relative odds ratio (ROR) and 95% confidence interval (CI); the probability was calculated using Fisher’s two-sided exact test. The level of significance was set at *p* ≤ 0.05.

## 3. Results

In our study, 16 different allele variants of the *HLA-G* gene were observed. In the studied population, the most common alleles were *10101* (47.48%) and *10102* (25.30%). The least common alleles in the Polish population were *10104*, *10109*, and *10402* (0.09% each) (Table 3). 

We analyzed the *HLA-G* allele frequencies in the parental pairs with antiphospholipid syndrome, preeclampsia, intrauterine growth restriction, and recurrent spontaneous abortion and in the control group (Table 4). The most common *HLA-G* allele in the groups of women with antiphospholipid syndrome, preeclampsia, intrauterine growth restriction, and recurrent spontaneous abortion was the *HLA-G 10101* allele found in 35.71%, 44.79%, 57.35%, and 52.59% of the women, respectively. In the control group, this allele was observed in 48.31% of the women.

Analysis of the *HLA-G* allele frequencies in the men―partners of the women with pregnancy complicated by antiphospholipid syndrome, preeclampsia, intrauterine growth restriction, and recurrent spontaneous abortion (Table 3)―demonstrated that the *HLA-G 10101* allele was the most common and was found in 45.37%, 47.67%, 47.14%, and 47.41% of the men, respectively, and in 51.74% of the men in the control group.

We also calculated an odds ratio (OR) for a complicated pregnancy depending on the *HLA-G* allele (Table 5). A significantly higher risk of the antiphospholipid syndrome in the women was associated with the *HLA-G 10108* allele (OR = 3.24; *p* = 0.042), while the *HLA*-G *10101* (OR = 0.59; *p* = 0.030) and the *HLA-G 10106* (OR = 0.21; *p* = 0.037) alleles reduced that risk. The women with the *HLA-G 10108* allele (OR = 5.91; *p* = 0.001) had almost a six-fold higher risk of preeclampsia than the control group. The *HLA-G* alleles in the women did not increase the risk of intrauterine growth restriction or recurrent spontaneous abortion. There was no relationship between the presence of the studied alleles in men and the risk of antiphospholipid syndrome, preeclampsia, intrauterine growth restriction, or recurrent spontaneous abortion in their partners.

The frequency of the *HLA-G* 14-bp ins/del polymorphism (women *p* > 0.101, men *p* > 0.336) in the control group met the Hardy–Weinberg equilibrium criteria. Table 6 shows the frequencies of the 14-bp ins/del polymorphism in the 3′-UTR of the *HLA-G* gene in women with antiphospholipid syndrome, preeclampsia, intrauterine growth restriction, and recurrent spontaneous abortion and their partners and in the control group. There were no statistically significant differences in the frequencies of the 14-bp ins/del polymorphism in the 3′-UTR of the *HLA-G* gene between the groups (*p* > 0.05) (Fisher’s two-sided exact test).

The lack of statistical significance (*p* > 0.05) was also noted in the analysis of the frequencies of the common *HLA-G* alleles and the common alleles of the 14-bp ins/del polymorphism in the 3′-UTR of the *HLA-G* gene in the parental pairs with a complicated pregnancy (Table 7).

## 4. Discussion

*HLA-G* is critical for effective implantation in pregnancy. Increasingly numerous scientific reports show that *HLA-G* plays an essential role in fetal tolerance through the mechanism of inhibition of the cytotoxic function of T and NK cells. The literature provides evidence that the polymorphic variants of the *HLA-G* antigen involve complications in pregnancy, such as recurrent spontaneous abortion and preeclampsia [15,16,17,18]. Nevertheless, the available studies compare various molecular changes in different populations and the reported data are often ambiguous.

In the pathogenesis of preeclampsia, the contribution of a wide range of genes interacting with environmental factors is analyzed. A high BMI, African American and South Asian races, chronic hypertension, pregnancy after ovulation induction therapy, a family or obstetric history of preeclampsia, as well as chronic hypertension and diabetes preceding pregnancy are factors that predispose to preeclampsia. It is suggested that genes associated with the function of the vascular endothelium, the function of the placenta, as well as blood coagulation and fibrinolysis may play a role [19,20].

Our investigation indicates that the risk of preeclampsia in female carriers of the *HLA-G 10108* allele is almost six-fold higher than in the control group. However, our analysis of the frequencies of the 14-bp ins/del polymorphism in the 3′-UTR of the *HLA-G* gene in particular groups have not demonstrated statistically significant differences compared with the control group. It is worth mentioning that in their study, Sipak-Szmigiel et al. [21] observed that an increased risk of failure in pregnancy (OR = 5.86; 95% CI; 0.93–21.24; *p* = 0.09) in the experimental group was associated with the frequency of the *HLA-G 10108* allele. Similar research was conducted by Abbas et al. [22], who analyzed the *HLA-G* polymorphisms in a group of 120 women with recurrent spontaneous abortion and 120 women with uncomplicated pregnancy in India. As the authors reported, the *HLA-G 010103* allele was more common in the women with recurrent spontaneous abortion and the *HLA-G 10105* allele was present in 1.7% of them. The *HLA-G 010108* allele was observed in 0.4% of the women with recurrent spontaneous abortion and was not found at all in the women with uncomplicated pregnancy. The frequency of the *HLA-G 0105N* allele in the Indian population was quite high (13.8%) compared with other worldwide populations. A similar study was carried out by Durmanova et al. [23], who analyzed 123 women with preeclampsia and 102 women with an uncomplicated pregnancy to assess potential links between the selected *HLA-G* polymorphisms and the risk of preeclampsia. Their results did not reveal any association between the *HLA-G* 14-bp polymorphism and the risk of preeclampsia. A similar observation was made by Rokhafrooz et al. [24], who performed genotyping of the 14-bp ins/del polymorphism in exon 8 of the *HLA-G* gene in 150 healthy pregnant women and 150 women with preeclampsia. In the study of Ferreira et al. [25] the polymorphism was not associated with the risk of developing preeclampsia [OR = 0.93 (0.72–1.19); *p* = 0.541], eclampsia [OR = 0.90 (0.60–1.38); *p* = 0.628], or HELLP (H—hemolysis, EL—elevated liver enzymes, LP—low platelets counts) syndrome [OR = 0.92 (0.66–1.28); *p* = 0.628]. Preeclampsia was not related to the *HLA-G* 14-bp genotype. On the contrary, Aldrich et al. reported no association between the HLA-G*0105N null allele and preeclampsia in African Americans [26], which was later confirmed in Caucasians in the study by Hylenius et al. [27]. Moreau et al. [28] and Tan et al. [29] also reported a significant association between the HLA-G*0106 allele and the risk of preeclampsia. Interestingly, the HLA-G*0106 allele includes the 14-bp *HLA-G* polymorphism. Yie et al. have reported a significant association between the SNP at position 1754 in exon 8 in the 3′-UTR of the *HLA-G* gene and the risk of preeclampsia [30]. In our study, the *HLA-G 106* allele (1.04%) was rare among women with previous episodes of preeclampsia and their risk of preeclampsia was low and statistically insignificant.

Our study did not confirm an increased risk of recurrent spontaneous abortion in the parental pairs with the *HLA-G* alleles. Kalotra et al. [8] analyzed the *HLA-G* 14-bp ins/del polymorphism in over 200 women with recurrent miscarriages (two or more miscarriages) and their partners, comparing it with the control group. They did not note significant differences in the frequencies of the *HLA-G* 14-bp ins/del polymorphism and the studied genotypes between the groups. The study of Hashemi et al. [31] provided evidence that the *HLA-G* 14-bp ins/del polymorphisms may contribute to the risk of recurrent spontaneous abortion. Nonetheless, our study did not confirm this relationship in the Polish population.

Another look at this issue was presented by Koc et al. in 2018 [32], who assessed the connection between the risk of miscarriage and the *HLA-G* polymorphism in the fetus. Similarly to us, they found that the most common *HLA-G* type, both in the study group (30.3%) and the control group (47%), was *HLA-G 10101*. They also reported that the *HLA-G 10104* allele was visibly related with miscarriage (*p* = 0.007), and the 14-bp deletion in the 3′-UTR of the *HLA-G* gene was more frequent in the group with miscarriages. The difference was not statistically significant.

Also, SNP mutations in the *HLA-G* promoter region are important from the point of view of maintaining pregnancy. Research in this field was conducted by Yazdani et al. [33], who investigated the –1573T> C and –1746C> A SNP mutations in the *HLA-G* promoter region, associated with recurrent spontaneous abortion. Their haplotype analysis revealed a significant link between recurrent miscarriages and the H1 (ATCCAGGTACGCAA) and H2 (CTTCGAGAACGCAG) haplotypes.

In our study, the higher risk of antiphospholipid syndrome for the women was accompanied by the presence of the *HLA-G 10108* allele, while the lower risk was related to the *HLA-G 10101* allele. The literature of the subject offers few publications describing the relationship between the *HLA-G* polymorphisms and antiphospholipid syndrome. Research on the contribution of the *HLA-G 10101*, *HLA-G 10108*, and *HLA-G 10106* alleles to the risk of complications in pregnancy in the Polish population requires further investigation.

## 5. Conclusions

Our study allows the conclusion that the *HLA-G 10108* allele may predispose women to antiphospholipid syndrome and preeclampsia. The *HLA-G 10101* and the *HLA-G 10106* alleles, on the other hand, may reduce the risk of antiphospholipid syndrome. The 14-bp ins/del polymorphism in the 3′-UTR of the *HLA-G* gene has most probably no effect on the risk of complications in pregnancy. The presence of the same *HLA-G* alleles in both partners probably does not modify the risk of complicated pregnancy.

### Limitations

One of the limitations of our study is that we did not control for risk factors of the outcome. However, we decided it was not necessary as our aim was to estimate the magnitude of causal effects. We would only have needed to control for risk factors if there was a systematic causal reason for the risk factor to be associated with the exposure variable in the underlying target population, which was not the case here. The risk factors for the pregnancy outcomes in our study were not a “cause” of the genotype. Those risk factors that might be unbalanced in exposure groups within the “sample” will be accounted for in the confidence intervals. They produce random error, not systematic error. A similar strategy has been described in the study of Hernán et al. [34], who claimed that analyses of the effects of prenatal exposures frequently adjust for variables, such as maternal weight gain during pregnancy, gestational age, or birth weight, that are likely to be affected by either the exposure or the outcome. The decision to adjust is usually based on statistical criteria only.

We also need to say that the almost six-fold increased odds of preeclampsia in female carriers is estimated with very low precision. Our 95% CI for that estimate is 2.1–16.3. As such, our standard errors are quite large. However, an odds ratio of <2.5 in the underlying “target” population is consistent with our findings. A large OR results from the lack of the studied alleles in particular groups.

## Figures and Tables

**Table 1:** Diagnostic criteria for pregnancy complicated by the antiphospholipid syndrome, preeclampsia, intrauterine growth restriction, or recurrent spontaneous abortion.

No.	Group	Number of Patients	Diagnostic Criteria
1	The group with antiphospholipid syndrome (APS)	70 women and their 54 partners	The syndrome was diagnosed on the basis of clinical criteria (the patient’s obstetric history, and/or experience of embolic/thrombotic complications, and/or autoimmune diseases), and laboratory criteria. The criteria for inclusion in the group were [10]: (1) Thrombosis—one or more episodes of arterial thrombosis, venous thrombosis (except for superficial venous thrombosis, SVT), or capillary thrombosis within any tissue or organ, confirmed by imaging, Doppler, or histological examination; (2) obstetric failure, i.e., at least one death of a morphologically normal fetus after the 10th week of pregnancy, or at least one premature birth of the morphologically normal fetus before the 34th week of pregnancy due to preeclampsia, eclampsia, or severe placental insufficiency, or at least three spontaneous miscarriages before the 10th week of pregnancy with the exclusion of anatomical causes and hormonal disorders in the mother, and chromosomal disorders in both parents; (3) laboratory criteria—the presence of lupus anticoagulant in plasma detected on at least two occasions minimum 12 weeks apart, using methods recommended by the International Society on Thrombosis and Haemostasis; mean or high levels of IgG or IgM class anticardiolipin antibodies (>40 GPL or MPL or <99th percentile) detected on at least two occasions minimum 12 weeks apart by a standardized ELISA method; anti-β2-glycoprotein I antibodies in serum or plasma (>99th percentile) detected on at least two occasions minimum 12 weeks apart.
2	The group with preeclampsia (PE)	48 women and their 43 partners	The criteria for a diagnosis of severe preeclampsia were [11,12]: (1) Increased blood pressure and proteinuria after the end of the 20th week of pregnancy (with the exception of gestational trophoblastic disease (GTD) and multifetal pregnancy); (2) the lack of proteinuria if the following occurred for the first time after the 20th week of pregnancy: Thrombocytopenia (a blood platelet count <100,000/µL), liver disease (two-fold higher transaminase activity than normal), impaired renal function (level of creatinine >1.1 mg/dL or a two-fold increase in the creatinine level without kidney disease in the patient’s medical history), pulmonary edema, central nervous system disorders, or vision disorders. The mean BMI of the pregnant women with preeclampsia was 28.1 (25.6–37.1); the mean birth weight of the infants was 2838.7 ± 514 g; 11 pregnant women were diagnosed with chronic hypertension. The patients after in vitro fertilization were not included in the study.
3	The group with intrauterine growth restriction (IUGR)	35 women and their 34 partners	In this group, a fetal weight was below the 10th percentile for the gestational age according to the first ultrasound performed in pregnancy, and the causes of intrauterine growth restriction—such as genetic determinants, diabetes, hypertension, preeclampsia, infections, renal disease, fetal developmental malformations, smoking, alcohol consumption, uterine abnormalities, taking medicines, autoimmune disease, etc.—were excluded.
4	The group with recurrent spontaneous abortion (RSA)	58 women and their 58 partners	The women in this group had at least three spontaneous miscarriages in the first trimester of pregnancy; other causes of miscarriages were excluded. In all pairs, a normal karyotype was confirmed and health problems, such as diabetes, thyroid and adrenal gland disease, anatomic abnormalities, TORCH infections (toxoplasmosis, other (syphilis, HIV, varicella), rubella, cytomegaloviral disease, herpes), other infections, and autoimmune disease (the presence of anticardiolipin antibodies, lupus anticoagulant, and antinuclear antibodies) were excluded.
5	The control group (C)	89 healthy women and their 86 partners	The women in this group had no significant perinatal history, had given birth at least twice, and their pregnancies were uncomplicated. In these women, embolic/thrombotic complications and concomitant autoimmune diseases were excluded.

**Table 2:** The nucleotide sequences of primers used in PCR and sequencing [13].

EXON	Sequence 5′→ 3′
EXON 2	F GGGTCGGGCGGGTCTCAA R TCCGTGGGGCATGGAGGT
EXON 3	F GGGGCTGACCGGGGGT R GCTAGGCCAGGCTGGGA
EXON 4	F CCATGAGAGATGCAAAGTGCT R TGCTTTCCCTAACAGACATGAT

**Table 3:** The frequency of the *HLA-G* alleles in the experimental and the control groups.

*HLA-G* Allele	Study Group		Control Group		Total	
	*n*	%	*n*	%	*n*	%
**105N**	13	1.63	6	1.71	19	1.65
**103**	5	0.63	0	0.00	5	0.43
**106**	40	5.00	11	3.14	51	4.43
**10101**	371	46.38	175	50.00	546	47.48
**10102**	202	25.25	89	25.43	291	25.30
**10103**	38	4.75	23	6.57	61	5.30
**10104**	1	0.13	0	0.00	1	0.09
**10105**	2	0.25	1	0.29	3	0.26
**10106**	9	1.13	7	2.00	16	1.39
**10107**	1	0.13	2	0.57	3	0.26
**10108**	59	7.38	14	4.00	73	6.35
**10109**	1	0.13	0	0.00	1	0.09
**10110**	12	1.50	2	0.57	14	1.22
**10401**	38	4.75	18	5.14	56	4.87
**10402**	0	0.00	1	0.29	1	0.09
**10403**	8	1.00	1	0.29	9	0.78
**Total**	800	100	350	100	1150	100

**Table 4:** The *HLA-G* allele frequencies in the women and men with antiphospholipid syndrome (APS), preeclampsia (PE), intrauterine growth restriction (IUGR), recurrent spontaneous abortion (RSA), and the control group (C).

*HLA-G* Allele	APS				PE				IUGR				RSA				C			
	Women *n* = 70		Men *n* = 54		Women *n* = 48		Men *n* = 43		Women *n* = 35		Men *n* = 34		Women *n* = 58		Men *n* = 58		Women *n* = 89		Men *n* = 86	
	*n*	%	*n*	%	*n*	%	*n*	%	*n*	%	*n*	%	*n*	%	*n*	%	*n*	%	*n*	%
**105N**	2	1.43	3	2.78	2	2.08	0	0.00	1	1.47	0	0.00	4	3.45	1	0.86	3	1.69	3	1.74
**103**	0	0.00	1	0.93	0	0.00	0	0.00	1	1.47	0	0.00	2	1.72	1	0.86	0	0.00	0	0.00
**106**	10	7.14	7	6.48	1	1.04	7	8.14	2	2.94	4	5.71	4	3.45	5	4.31	5	2.81	6	3.49
**10101**	50	35.71	49	45.37	43	44.79	41	47.67	39	57.35	33	47.14	61	52.59	55	47.41	86	48.31	89	51.74
**10102**	38	27.14	25	23.15	26	27.08	22	25.58	13	19.12	23	32.86	25	21.55	30	25.86	50	28.09	39	22.67
**10103**	7	5.00	5	4.63	4	4.17	2	2.33	4	5.88	3	4.29	5	4.31	8	6.90	11	6.18	12	6.98
**10104**	0	0.00	0	0.00	0	0.00	0	0.00	0	0.00	0	0.00	0	0.00	1	0.86	0	0.00	0	0.00
**10105**	0	0.00	0	0.00	1	1.04	0	0.00	0	0.00	0	0.00	0	0.00	1	0.86	0	0.00	1	0.58
**10106**	0	0.00	2	1.85	1	1.04	1	1.16	1	1.47	2	2.86	1	0.86	1	0.86	6	3.37	1	0.58
**10107**	1	0.71	0	0.00	0	0.00	0	0.00	0	0.00	0	0.00	0	0.00	0	0.00	0	0.00	2	1.16
**10108**	12	8.57	5	4.63	14	14.58	9	10.47	4	5.88	2	2.86	8	6.90	5	4.31	5	2.81	9	5.23
**10109**	1	0.71	0	0.00	0	0.00	0	0.00	0	0.00	0	0.00	0	0.00	0	0.00	0	0.00	0	0.00
**10110**	3	2.14	3	2.78	2	2.08	0	0.00	0	0.00	2	2.86	1	0.86	1	0.86	1	0.56	1	0.58
**10401**	13	9.29	5	4.63	2	2.08	4	4.65	2	2.94	0	0.00	5	4.31	7	6.03	9	5.06	9	5.23
**10402**	0	0.00	0	0.00	0	0.00	0	0.00	0	0.00	0	0.00	0	0.00	0	0.00	1	0.56	0	0.00
**10403**	3	2.14	3	2.78	0	0.00	0	0.00	1	1.47	1	1.43	0	0.00	0	0.00	1	0.56	0	0.00
**Total**	140	100	108	100	96	100	86	100	68	100	70	100	116	100	116	100	178	100	172	100

**Table 5:** The *HLA-G* allele frequencies in the women with antiphospholipid syndrome (APS), preeclampsia (PE), intrauterine growth restriction (IUGR), recurrent spontaneous abortion (RSA), and their partners. The risk of APS, PE, IUGR, and RSA in these women and in the control group (C).

***HLA-G*** **Allele**	**APS vs. C**						**PE vs. C**					
	**Women**			**Men**			**Women**			**Men**		
	**OR**	**(95% Cl)**	***p***	**OR**	**(95% Cl)**	***p***	**OR**	**(95% Cl)**	***p***	**OR**	**(95% Cl)**	***p***
**105N**	0.85	0.00–4.30	1.000	1.61	0.36–7.11	0.679	1.24	0.00–6.34	1.000	0.00	0.00–2.56	0.553
**106**	2.66	0.93–7.62	0.108	1.92	0.66–5.60	0.258	0.36	0.00–2.40	0.669	2.45	0.83–7.20	0.133
**10101**	0.50	0.38–0.93	0.03	0.77	0.48–1.25	0.327	0.87	0.53–1.34	0.613	0.85	0.51–1.42	0.598
**10102**	0.95	0.58–1.56	0.9	1.03	0.58–1.81	1.000	0.95	0.55–1.65	0.889	1.17	0.65–2.13	0.642
**10103**	0.80	0.31–2.06	0.808	0.65	0.23–1.82	0.608	0.66	0.22–2.03	0.586	0.32	0.00–1.30	0.152
**10106**	0.21	0.00–0.80	0.037	3.23	0.42–	0.561	0.3	0.00–1.95	0.427	2.01	0.00–	1.000
**10108**	3.24	1.16–9.05	0.042	0.88	0.30–2.58	1.000	5.91	2.13–16.29	0.001	2.12	0.83–5.39	0.128
**10110**	3.88	0.55–	0.324	4.89	0.69–	0.162	3.77	0.49–	0.282	0.99	0.00–	1.000
**10401**	1.92	0.81–4.53	0.182	0.88	0.30–2.58	1.000	0.4	0.00–1.68	0.339	0.88	0.28–2.88	1.000
***HLA-G*** **Allele**	**IUGR vs. C**						**RSA vs. C**					
	**Women**			**Men**			**Women**			**Men**		
	**OR**	**(95% Cl)**	***p***	**OR**	**(95% Cl)**	***p***	**OR**	**(95% Cl)**	***p***	**OR**	**(95% Cl)**	***p***
**105N**	0.87	0.00–6.23	1.000	0.82	0.00–3.15	0.559	2.08	0.51–8.48	0.44	0.49	0.00–3.48	0.651
**106**	1.05	0.00–4.83	1	1.68	0.49–5.73	0.481	1.24	0.35–4.35	0.743	1.25	0.39–3.95	0.761
**10101**	1.44	0.82–2.52	0.254	0.83	0.48–1.45	0.572	1.19	0.74–1.89	0.551	0.84	0.53–1.35	0.548
**10102**	0.61	0.31–1.19	0.191	1.67	0.91–3.07	0.107	0.7	0.41–1.22	0.221	1.19	0.69–2.05	0.575
**10103**	0.95	0.31–2.94	1.000	0.6	0.18–2.04	0.564	0.68	0.24–1.94	0.604	0.99	0.40–2.44	1.000
**10106**	0.43	0.00–2.78	0.677	5.03	0.65–	0.202	0.25	0.00–1.16	0.251	1.49	0.00–	1.000
**10108**	2.16	0.61–7.70	0.266	0.53	0.00–2.26	0.518	2.56	0.86–7.64	0.144	0.82	0.28–2.39	0.787
**10110**	0.00	0.00–	1.000	5.03	0.65–	0.202	1.54	0.00–12.61	1.000	1.49	0.00–	1.000
**10401**	0.57	0.00–2.41	0.732	0.26	0.00–1.01	0.063	0.85	0.29–2.48	1.000	1.16	0.44–3.11	0.797

Fisher’s two-sided exact test.

**Table 6:** The frequencies of the 14-bp ins/del polymorphism in the 3′-UTR of the *HLA-G* gene in the women with antiphospholipid syndrome, preeclampsia, intrauterine growth restriction, recurrent spontaneous abortion and their partners and in the control group.

**14-bp ins/del Genotype**										
**Polymorphism in Women**	**APS** ***n* = 70**		**PE** ***n* = 48**		**IUGR** ***n* = 35**		**RSA** ***n* = 58**		**C** ***n* = 89**	
	***n***	**%**	***n***	**%**	***n***	**%**	***n***	**%**	***n***	**%**
ins/del	24	34.29	19	39.58	15	44.12	22	37.93	33	37.08
del/del	31	44.29	21	43.75	16	47.06	27	46.55	42	47.19
ins/ins	15	21.43	8	16.67	3	8.82	9	15.52	14	15.73
**Polymorphism in Men**	**APS** ***n* = 54**		**PE** ***n* = 43**		**IUGR** ***n* = 34**		**RSA** ***n* = 58**		**C** ***n* = 86**	
	***n***	**%**	***n***	**%**	***n***	**%**	***n***	**%**	***n***	**%**
ins/del	17	31.48	13	30.23	11	31.43	23	39.66	34	39.53
del/del	30	55.56	23	53.49	18	51.43	27	46.55	41	47.67
ins/ins	7	12.96	7	16.28	6	17.14	8	13.79	11	12.79

**Table 7:** The frequencies of the *HLA-G* common alleles and the 14-bp ins/del polymorphism in the 3′-UTR of the *HLA-G* gene in the parental pairs with a pregnancy complicated by antiphospholipid syndrome, preeclampsia, intrauterine growth restriction, or recurrent spontaneous abortion and in the control group.

Allele	Allele Common for Partners	APS *n* = 53		PE *n* = 39		IUGR *n* = 32		RSA *n* = 58		C *n* = 80	
		*n*	%	*n*	%	*n*	%	*n*	%	*n*	%
**HLA-G**	none	21	39.62	19	15.00	15	11.00	22	37.93	10	17.54
	one	27	50.94	21	18.00	16	18.00	27	46.55	44	77.19
	both	5	9.43	8	6.00	3	3.00	9	15.52	3	5.26
**14-bp ins**	none	12	22.64	6	15.38	4	12.50	9	15.52	12	15.00
	one	21	39.62	19	48.72	13	40.63	29	50.00	46	57.50
	both	20	37.74	14	35.90	15	46.88	20	34.48	22	27.50
